# An Inverse Switch in DNA Base Excision and Strand Break Repair Contributes to Melphalan Resistance in Multiple Myeloma Cells

**DOI:** 10.1371/journal.pone.0055493

**Published:** 2013-02-06

**Authors:** Mirta M. L. Sousa, Kamila Anna Zub, Per Arne Aas, Audun Hanssen-Bauer, Aida Demirovic, Antonio Sarno, Erming Tian, Nina B. Liabakk, Geir Slupphaug

**Affiliations:** 1 Department of Cancer Research and Molecular Medicine, Norwegian University of Science and Technology (NTNU), Trondheim, Norway; 2 The Proteomics and Metabolomics Core Facility (PROMEC), Norwegian University of Science and Technology (NTNU), Trondheim, Norway; 3 The KG Jebsen Center for Myeloma Research, Norwegian University of Science and Technology (NTNU), Trondheim, Norway; 4 Laboratory of Myeloma Genetics, Myeloma Institute for Research and Therapy, University of Arkansas for Medical Sciences, Little Rock, Arkansas, United States of America; Texas Tech University, United States of America

## Abstract

Alterations in checkpoint and DNA repair pathways may provide adaptive mechanisms contributing to acquired drug resistance. Here, we investigated the levels of proteins mediating DNA damage signaling and -repair in RPMI8226 multiple myeloma cells and its Melphalan-resistant derivative 8226-LR5. We observed markedly reduced steady-state levels of DNA glycosylases UNG2, NEIL1 and MPG in the resistant cells and cross-resistance to agents inducing their respective DNA base lesions. Conversely, repair of alkali-labile sites was apparently enhanced in the resistant cells, as substantiated by alkaline comet assay, autoribosylation of PARP-1, and increased sensitivity to PARP-1 inhibition by 4-AN or KU58684. Reduced base-excision and enhanced single-strand break repair would both contribute to the observed reduction in genomic alkali-labile sites, which could jeopardize productive processing of the more cytotoxic Melphalan-induced interstrand DNA crosslinks (ICLs). Furthermore, we found a marked upregulation of proteins in the non-homologous end-joining (NHEJ) pathway of double-strand break (DSB) repair, likely contributing to the observed increase in DSB repair kinetics in the resistant cells. Finally, we observed apparent upregulation of ATR-signaling and downregulation of ATM-signaling in the resistant cells. This was accompanied by markedly increased sensitivity towards Melphalan in the presence of ATR-, DNA-PK, or CHK1/2 inhibitors whereas no sensitizing effect was observed subsequent to ATM inhibition, suggesting that replication blocking lesions are primary triggers of the DNA damage response in the Melphalan resistant cells. In conclusion, Melphalan resistance is apparently contributed by modulation of the DNA damage response at multiple levels, including downregulation of specific repair pathways to avoid repair intermediates that could impair efficient processing of cytotoxic ICLs and ICL-induced DSBs. This study has revealed several novel candidate biomarkers for Melphalan sensitivity that will be included in targeted quantitation studies in larger patient cohorts to validate their value in prognosis as well as targets for replacement- or adjuvant therapies.

## Introduction

Multiple myeloma (MM) is a clonal B-cell malignancy characterized by abnormal proliferation of malignant plasma cells in the bone marrow, leading to impaired hematopoiesis as well as osteolytic bone destruction [1]. As a consequence, MM patients often experience bone pain, bone fractures, hypercalcemia and fatigue. In addition, MM cells produce excessive amounts of non-functional antibodies, which mediate increased susceptibility to infections. MM is the second most prevalent haematological malignancy (approximately 10%) following non-Hodgkin’s lymphoma and constitute about 1% of all malignancies. It is also showing substantial and systematic mortality (1% of total cancer deaths) in the elderly of most areas worldwide [1], [2].

Since its introduction in 1958, Melphalan (L-phenylalanine mustard, Alkeran, CAS 148-82-3) [3] has been a common agent to treat MM. In combination with prednisone (MP) it has been the core treatment for patients with newly diagnosed MM who are not eligible for autologous stem cell transplant (ASCT) and is also central in high dose therapy (HDM) prior to ASCT [4], [5]. More recently, MP has also been combined with novel agents such as thalidomide, lenalidomide and bortezomib in patients not eligible for ASCT [6] and this has increased survival ([7] and references therein). Although the initial response to Melphalan-based treatment is generally good, treatment is limited by development of acquired drug resistance (ADR) [8] and eventually all cases become refractive [6]. There is thus an urgent need to develop means for early detection of ADR to improve prognosis and treatment.

Melphalan is a bifunctional alkylating agent belonging to the nitrogen mustard class of chemotherapeutic agents, and induces both DNA monoadducts and ICLs [9], [10]. Although ICLs apparently constitute a minor fraction of the DNA lesions introduced by Melphalan [11], [12] they have been regarded the major cytotoxic lesions since they block DNA replication and induce the formation of DNA double-strand breaks (DSBs) [9]. Modulation of several cellular processes have been suggested to contribute to resistance, including reduced drug uptake due to defective drug transport [13], elevated glutathione levels [14], [15], decreased apoptosis [16], [17] and modulated interaction of the myeloma cells with extracellular matrix [18]. More recently, modulation of DNA damage signaling- and repair pathways have also been suggested to be major contributors to Melphalan resistance [19]–[21]. This work has primarily focused upon the role of the Fanconi Anemia (FA)/BRCA1 pathway in enhancing ICL repair, thereby promoting cell survival [17], [21]–[24]. However, Melphalan may form a number of different adducts in DNA, including (but likely not limited to) intra- and interstrand guanine N7 crosslinks, adenine N3 alkylations and DNA-protein crosslinks [25], [26]. Moreover, a marked elevation of oxidative stress markers has been observed in patients undergoing MP-based conditioning regimen prior to ACST, as well as subsequent to transplant [27] suggesting that oxidative DNA damage may also contribute to cytotoxicity. The quantitative contribution of each type of DNA lesion to Melphalan cytotoxicity remains, however, to be established.

Repair of ICLs is not yet fully understood. However, proteins belonging to several different DNA repair pathways cooperate to resolve these complex lesions, including proteins contributing to the FA-pathway, nucleotide excision repair (NER), mismatch repair (MMR) and double-strand break repair (DSBR) ([28] and references therein). More recently, the involvement of base excision repair (BER) in development of resistance to DNA crosslinkers has been increasingly recognized [29], [30]. Interestingly, while enhanced BER activities mediate resistance against antitumor agents in some types of cancer [31]–[33], BER deficiency has also been connected with development of Cisplatin resistance [34]. To gain more insight into potential contribution to Melphalan resistance by proteins involved in DNA damage signaling and repair, we investigated the expression of a broad panel of proteins involved in the DNA damage response in the Melphalan-sensitive multiple myeloma cell line RPMI8226 and its Melphalan resistant derivative 8226-LR5. Interestingly, our findings demonstrate that expression of several factors not previously associated with Melphalan resistance is altered in the resistant cells. Specifically we observed a striking downregulation of several DNA glycosylases in the resistant cells in parallel with apparently increased efficiency of single- (SSB) and double strand break (DSB) repair. This suggest that resistant cells modulate the repair pathways to avoid activation of multiple stress response mechanisms that could interfere with processing of highly cytotoxic ICLs and DSBs.

The present results contribute novel candidate factors to be included in targeted protein profiling analyses by e.g. high-throughput MRM-mass spectrometry of patient samples to further evaluate their prognostic value. In addition, the apparent Melphalan-induced downregulation of specific BER proteins may provide clues adjuvant therapies to overcome Melphalan resistance.

## Results and Discussion

### Melphalan Resistance in RPMI8226 Cells is Accompanied by Increased S-phase Progression and Reduced G2/M Arrest Compared to Melphalan Sensitive Parental Cells

**Figure 1:**
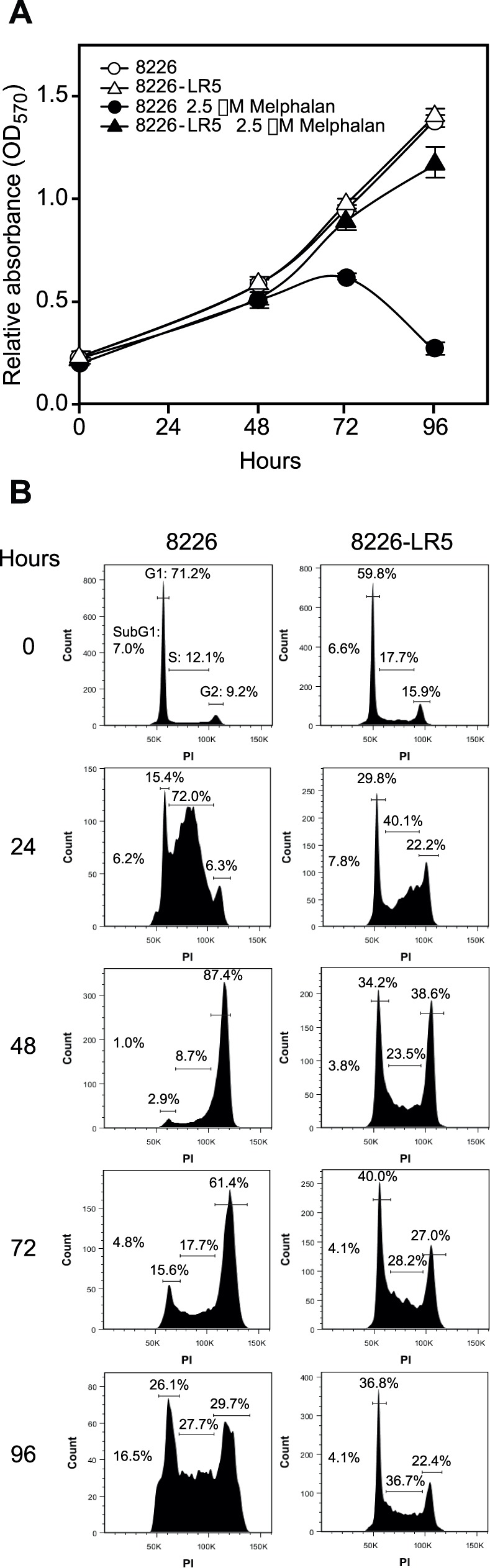
Melphalan resistance in RPMI8226 cells is accompanied by increased S-phase progression and reduced G2/M arrest. Cell growth rate (A) and cell cycle distribution (B) of sensitive (8226) and resistant (LR5) cells exposed or non-exposed to 2.5 µM Melphalan. (A) Exposure to 2.5 µM Melphalan greatly reduced the proliferation of the sensitive cells, while a minor effect upon the proliferation of resistant cells was observed. (B) Flow cytometric analysis of cell cycle phases shows that 2.5 µM Melphalan caused delayed S-phase progression in sensitive cells followed by G2/M arrest. Conversely, progression throughout S-phase was less affected in the resistant cells exposed to Melphalan. Moreover, the resistant cells were able to overcome the G2/M arrest induced by Melphalan and re-enter the cell cycle. Per cent cells in each of the cell cycle phases are indicated above the histograms. The fraction of cells in subG1 is indicated, but not illustrated in the gated histograms.

To mimic Melphalan concentrations observed in plasma of patients receiving oral Melphalan treatment [35], [36] and to maintain similar growth rate of the sensitive/resistant cell lines, the LR5 cells were added Melphalan to a final concentration of 1 µM at each passage. At this drug concentration we observed essentially identical growth rate (Fig. 1A) and similar cell cycle distribution (Fig. 1B, 0 hrs) of LR5 compared to the unexposed, parental 8226 cells (referred to as steady state growth conditions in the following). This is important when DNA repair proteins are the subject of analysis, since the expression of many proteins involved in DNA damage signaling and repair are strictly cell cycle regulated [37]–[43]. Whereas addition of Melphalan to a final concentration of 2.5 µM to both cell lines mediated a weak reduction of the proliferation of the resistant LR5 cells, proliferation of the sensitive cells essentially ceased 3 days after Melphalan addition (Fig. 1A). Flow cytometry analysis indicated that Melphalan exposure mediated a markedly delayed S-phase progression in the sensitive- compared to the resistant cells, followed by accumulation of cells in G2 (Fig. 1B). This delayed S-phase progression conforms to introduction of DNA lesions that delay or stall replication forks, as well as to potential accumulation of unrepaired damage that activates the G2/M checkpoint and induces apoptosis. This is supported by a markedly increased sub-G1 fraction among the sensitive cells from 72 to 96 hours post Melphalan addition, concomitant with a selective loss of cells in G2 (Fig. 1B, bottom panels). Conversely, S-phase progression was much less affected in the resistant LR5 cells, in agreement with a more efficient removal or bypass of replication-blocking DNA lesions, potentially accompanied with less efficient intra-S and G2/M checkpoints.

### Melphalan Resistance in RPMI8226 Cells is Accompanied by Modulated Expression of Proteins Belonging to Several DNA Damage Response Pathways

**Figure 2:**
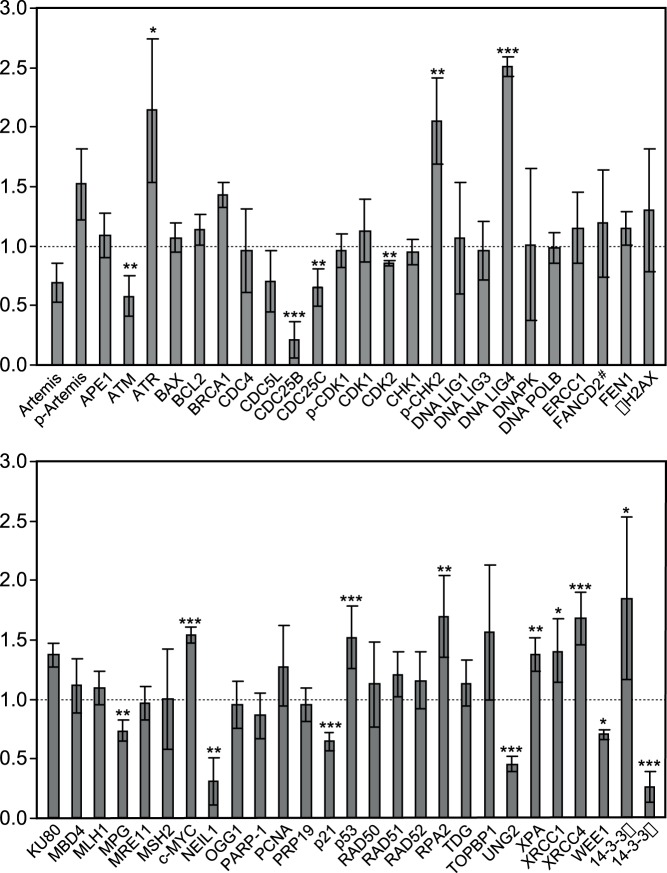
Steady state expression levels of proteins involved in DNA repair and DNA damage signaling response in 8226-LR5 cells relative to parental cells. Protein levels were assessed by quantitative western blot analysis using specific antibodies against target proteins. Each bar represent the mean expression ratio of the target protein in the LR5 (resistant) cells relative to the reference value of 1 (dotted line) in the sensitive cells subsequent to normalization against either β-actin or tubulin (to avoid overlapping signals). Quantitative analysis enclosed an average of 3 to 5 biological replicates with standard deviations as indicated. The P values were calculated by one sample two tailed t test against a hypothetical expression ratio set to 1 (no change in expression). >90%, >95% and >98% confidence levels indicated by *, **, and ***, respectively. # Only non-ubiquitinylated FANCD2 was detected in the analyses.

To investigate the molecular mechanisms underlying Melphalan resistance in the adapted LR5 cell line in more detail, and the potential role of DNA-repair and signaling proteins, we performed a quantitative western analysis of a panel of more than 50 proteins directly and/or indirectly involved in the DNA damage response. We chose western analysis since many of the involved proteins exist in the cells at levels that precluded a broad quantitative mass spectrometry profiling by our current instrumentation unless pre-enrichment strategies were included. Protein levels were analyzed both during continuous growth of the sensitive/resistant cells, as well as subsequent to a 6 h exposure to high-dose Melphalan (50 µM), corresponding to the lag-period reported where the conversion of mono-adducts into ICLs reaches a maximum [15]. Expression levels in the Melphalan resistant LR5 cells relative to the sensitive parental cells under continuous growth are summarized in Fig. 2, and demonstrate marked changes in the steady-state levels of several proteins belonging to different DNA damage signaling- and repair pathways. Most notably, we observed upregulation of the DNA damage sensory kinase ATR in the resistant cells with a concomitant decrease in ATM, indicating increased capability to detect and process replication-blocking lesions in the resistant cells. Moreover, several proteins involved in DSB repair by NHEJ were found to be upregulated in the resistant cells, most notably LIG4 and XRCC4. Conversely, three BER glycosylases (NEIL1, UNG2 and MPG) were found to be downregulated, indicating a reduced base excision capacity in the resistant cells. Somewhat surprisingly, we found no apparent up-regulation of the FA-pathway in the LR5 cells, as determined from the total level of FANCD2 as well as the apparent absence of monoubiquitinylated FANCD2 (Fig. 2). Ub-FANCD2 is considered an essential link between the FA protein complex and the BRCA1 repair machinery [44], and has previously been reported to be modestly upregulated in LR5 cells adapted to growth in 5µM Melphalan [24]. However, in another multiple myeloma cell line, U266, no increase in FANCD2 was observed subsequent to growth adaption to 6 µM Melphalan [17].

Among the cell-cycle and growth regulatory proteins we found that the oncogenes c-MYC and 14-3-3β were upregulated in the resistant cells, while Cdc25B/C and the tumor suppressor 14-3-3σ are markedly downregulated in the same cells. 14-3-3σ is a known inhibitor of G2/M-progression in many cell types subsequent to DNA damage [45], while 14-3-3β has been shown to stimulate proliferation [46]. The markedly shifted expression of these 14-3-3 proteins might thus contribute to the apparently reduced G2/M checkpoint-activation in the resistant cells.

### Melphalan Resistant Cells have Reduced Expression of Several BER-initiating Glycosylases and Display Cross-resistance to Agents that Mediate Non-bulky DNA Base Alterations

**Figure 3:**
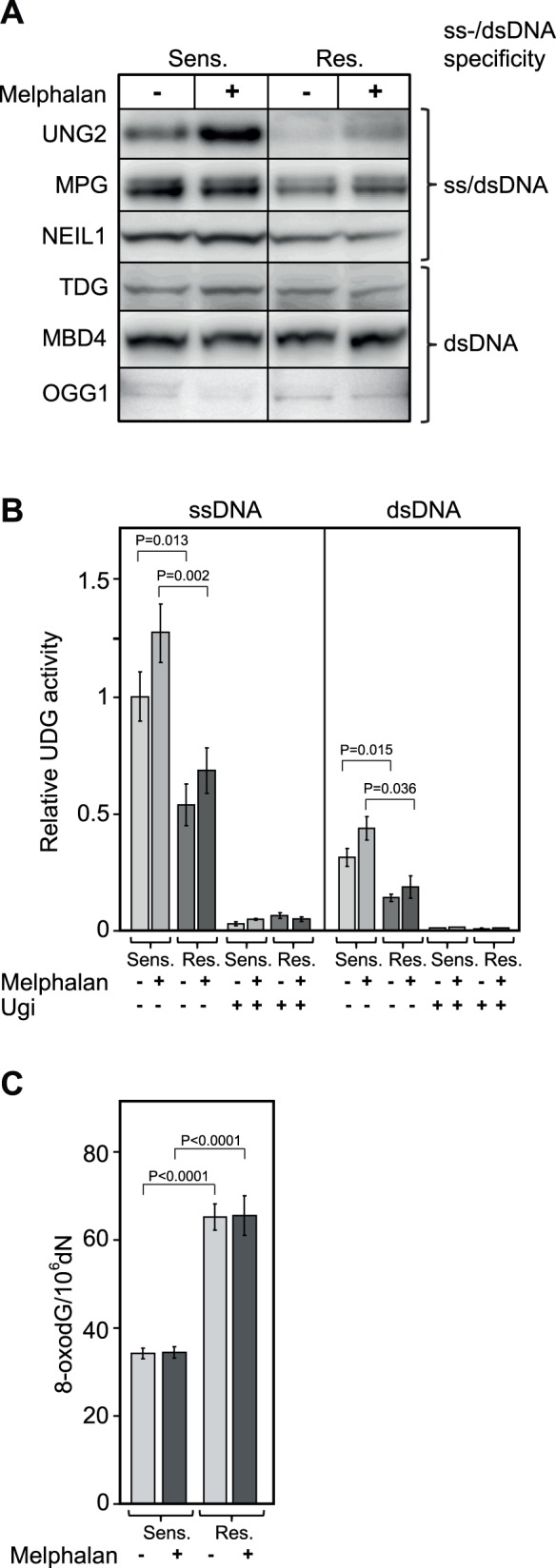
DNA glycosylases with dual ss/dsDNA substrate specificity are downregulated in Melphalan-resistant cells. Levels of DNA glycosylases (A), relative UDG activities (B) and genomic 8-oxodG-content in sensitive and resistant cells prior and after exposure to 50 µM Melphalan. Western blot analysis revealed downregulation of UNG2, MPG and NEIL1 (dual ss/dsDNA-specific glycosylases) in the Melphalan-resistant cells compared to sensitive cells while this was not observed for the dsDNA-specific glycosylases TDG, MBD4 and OGG1, the latter barely above the detection level in the cells (A). In agreement with UNG2 protein levels, activity assays showed that uracil excision activity was significantly higher in sensitive cells against [^3^H]dUMP-containing ss- and dsDNA substrates. Moreover, high dose Melphalan mediated an apparent increase in UDG activity in both cell lines, although this was only significant (P<0.05) for the resistant cells. Addition of the UNG-specific inhibitor Ugi to the reactions demonstrated that the observed reduced uracil-excision activity in the resistant cell line was mainly accounted for by reduction of UNG-activity. Each bar constitutes an average from four independent experiments, each run in triplicate, with standard deviations as demonstrated. P-values were calculated using unpaired Student t-test (B). A twofold increased steady state level of genomic 8-oxodG was found in the resistant compared to the sensitive cells. Each bar constitutes and average from three independent experiments with standard deviations as indicated (C).

BER is the dominating cellular pathway for repair of non-bulky DNA base damage [47]. A function of BER in the repair of Melphalan-induced DNA damage is thus not obvious, unless secondary lesions are induced by yet unidentified mechanisms. One exception is, however, the finding that the DNA glycosylase NEIL1 is able to excise a bulky unhooked psoralen-induced ICL by cleavage of the glycosidic bond between the deoxyribose and the crosslinked base [48] Surprisingly, however, we observed a marked downregulation at steady state of 3 out of 6 initiator glycosylases of BER (Fig. 3 A) in the LR5 cells whereas levels of downstream (damage- general) enzymes (APE1, POLB, FEN1, LIG1 and LIG3) remained unaltered (Fig. 2). Interestingly, the downregulated glycosylases NEIL1 (0.30±0.20), UNG2 (0.45±0.06) and MPG (0.73±0.09), share a common characteristic in that they are able to excise their respective base substrates (oxidised pyrimidines, uracil, and alkylated bases, respectively) from both dsDNA- as well as ssDNA contexts. Conversely, the levels of TDG, MBD4 and OGG1 that are all strictly dsDNA-specific, were essentially unaltered in the LR5 cells compared to the parental 8226 cells. Subsequent to a 6 h treatment with 50 µM Melphalan, UNG2 was upregulated in both the sensitive and resistant cells whereas little modulation of the protein levels of the other glycosylases was observed (Fig. 3A). Since UNG2 is strictly cell-cycle regulated with the highest expression in late G1/S-phase [40], [49], the most likely explanation for the apparent upregulation is likely accumulation in S-phase of cells at the high (50 µM) concentration of Melphalan used in the experiment. This was also supported by the more pronounced upregulation in the sensitive cells which conforms to the strong S-phase accumulation subsequent to Melphalan treatment (Fig. 1B). The reduced level of UNG2 protein was also accompanied by a reduced uracil-excision activity in resistant cell free extracts and in both cell extracts this activity increased subsequent to acute exposure to 50 µM melphalan (Fig. 3B). Moreover, the increased activity was significant (P<0.05) in the sensitive cells only, in agreement with the UNG2 western analysis. Repeating the excision experiments in presence of the UNG-specific inhibitor Ugi encoded by the bacteriophage PBS2 [50] demonstrated that the overall contribution from other glycosylases than UNG to the total uracil-excision activity was <10% in both cell lines (Fig. 3B). Thus the reduced uracil-excision capacity in the resistant cells was mediated primarily by reduced level of UNG protein.

To monitor whether the markedly reduced level of NEIL1 as well as the low level of OGG1 (barely above detection level, Fig. 3A) was reflected in the genomic content of genomic oxidative damage, we analyzed the genomic 8-oxodG in both cell lines at steady state as well as 6 h subsequent to acute 50 µM Melphalan exposure. As illustrated in Fig. 3C, the steady state 8-oxodG level in the resistant cells was about twofold higher than that of the sensitive cells. Remarkably, however, acute exposure to 50 µM did not mediate any increase in 8-oxodG in any of the cell lines. To investigate the underlying cause of genomic 8-oxodG accumulation in the resistant cell line in more detail, we also monitored the total level of reactive oxygen species (ROS) in the two cell lines. We observed about 20% lower ROS level in the resistant- compared to the sensitive cells, but no significant difference in any of the cell lines after treatment with 50 µM Melphalen. In contrast, treatment with 50 µM of the positive control tert-butyl hydroperoxide (TBHP) mediated about threefold increased ROS in both cell lines (Fig. S1). The most likely interpretation of these results is that the markedly increased accumulation of genomic 8-oxodG in the resistant cells is not primarily caused by Melphala-induced ROS, but more likely result from lack of removal of 8-oxodG formed by endogenous sources of ROS. This would conform to the apparently very low level of OGG1 in both cell lines as well as the markedly (threefold) downregulated level of NEIL1 in the resistant cells.

**Figure 4:**
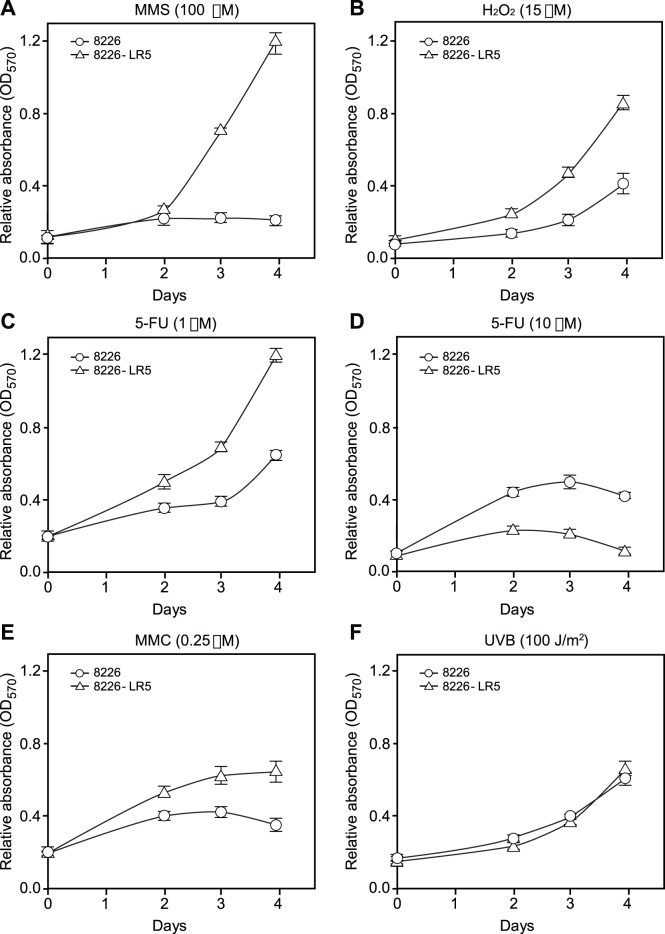
Melphalan resistance is accompanied by increased resistance to agents inducing non-bulky BER substrates. Cell were exposed to MMS (A), H_2_O_2_ (B), low (C) and high (D) doses of 5-FU, MMC (E) and UVB (F) assessed by the MTT assay. Viability curves showed that the Melphalan-resistant cells are cross-resistant to MMS, H_2_O_2_, low dose 5-FU and MMC. Conversely, high dose of 5-FU affects the proliferation of resistant cells in higher extent compared to sensitive cells. Exposure to UVB similarly affected growth of both cell lines. Increasing absorbance correlates directly with the number of living cells. Standard deviation bars are indicated.

Next we investigated whether the reduced levels of DNA glycosylases were accompanied by increased sensitivity towards agents inducing BER substrate lesions. To this end we subjected the cells to treatment with the alkylator methyl methanesulfonate (MMS), H_2_O_2_ and the antimetabolite 5-fluorouracil (5-FU). The latter induces both genomic uracil and 5-FU, both of which are substrates for UNG2 (reviewed in [51]). In addition, we monitored potential cross-resistance towards the ICL-inducing agent mitomycin C (MMC) and UVB-irradiation (Fig. 4). Notably, the Melphalan-resistant cells displayed a markedly increased resistance to both MMS and H_2_O_2_ (Fig. 3A,B). This was rather surprising, given the observed reduced levels of the respective glycosylases (MPG and NEIL1) that excise the major base lesions induced by these agents [52]–[53]. A similar cross-resistance was observed with low concentrations (1 µM) of 5-FU, whereas at high concentrations (10 µM) the Melphalan-sensitive cells displayed higher resistance to 5-FU (Fig. 4 B,C). Finally, the Melphalan-resistant cells displayed cross-resistance to the ICL-inducing agent MMC (Fig. 4E) whereas both cell lines were equally sensitive to UVB (Fig. 3F). The latter suggests that modulation of the expression of NER-factors does not likely contribute to the Melphalan-resistant phenotype in the 8226 cells.

The apparently increased tolerance of the Melphalan-resistant cell line towards agents causing structurally minor DNA-base-lesions (BER substrates) was intriguing. Previous studies have, however, demonstrated that overexpression of MPG mediates increased sensitivity towards MMS [54], likely because the number of AP-sites generated exceeds the capacity of downstream BER-factors and results in increased levels of cytotoxic repair intermediates. Furthermore, it was recently demonstrated that UNG-deficient cells displayed a Cisplatin-resistant phenotype accompanied by enhanced repair of ICLs and ICL-induced DSBs [34]. The authors demonstrated that (extrahelical) cytosines flanking the Cisplatin-ICL underwent preferential oxidative deamination *in vitro* and proposed that UNG-mediated removal of such flanking uracils induce AP-sites and BER intermediates than compete with productive ICL DNA repair. Whether such preferential deamination of cytosine also occurs at Melphalan-ICLs remains, however, to be established. Nevertheless, since UNG2, NEIL1 and MPG are major BER initiators of endogenous DNA base damage, decreased steady-state levels of BER repair intermediates (AP-sites and single-strand breaks (SSBs)) could mediate a survival benefit during the more complex processing of Melphalan-induced DNA damage.

### Melphalan-Resistance is Accompanied by Reduced Level of Genomic Alkali-labile Sites (ALS) and Increased Sensitivity to PARP-1 Inhibition

**Figure 5:**
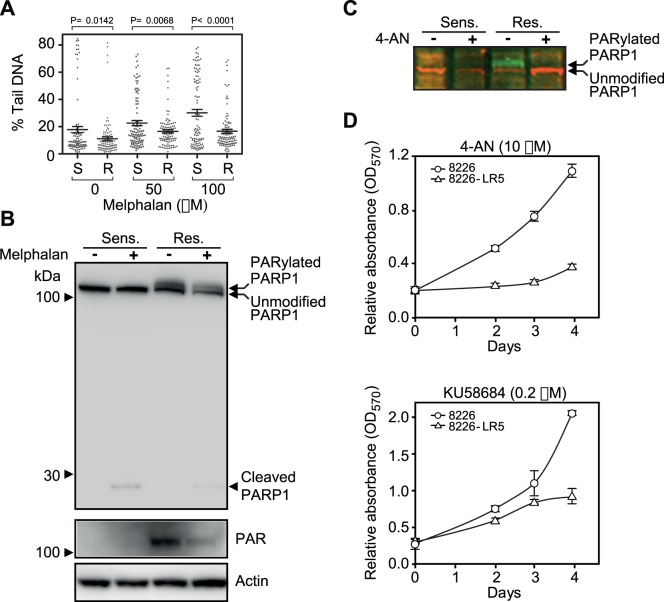
Assessment of alkali-labile sites in Melphalan sensitive and resistant cells. (A) Comet assays demonstrated that the resistant LR cells contained a significantly lower number of alkali-labile sites than the sensitive cells both at steady-state and subsequent to high-dose melphalan treatment. Per cent tail DNA of the comets is presented in dot plots with average and corresponding SEM. P values were calculated using an unpaired two-tailed t-test. R: resistant, S: sensitive. 100 comets were randomly selected and evaluated from one experiment at each treatment. (B) Western blot analysis showed that PARP-1 is activated via poly-ADP(ribosyl)ation only in the resistant cells suggesting that SSB repair and potentially also DSB synapsis is enhanced in the resistant cells. (C) Activation of PARP1 by PARylation in the resistant LR5 cells was completely abolished subsequent to treatment with the PARP-1 inhibitor 4-AN. (D) Inhibition of PARP-1 by 4-AN or KU58684 obstructs the proliferation of resistant cells while having a minor effect on the sensitive cells.

To investigate whether the reduced levels of BER glycosylases was also accompanied modulated levels of ALS, including AP-sites and SSBs, we performed an alkaline comet assay of both cell lines in the absence of- and subsequent to high dose (50 µM and 100 µM) Melphalan. Notably the resistant cells displayed significantly lower values of tail DNA both in the absence- and presence of high dose Melphalan (Fig. 5A) in agreement with the decreased level of initiator BER glycosylases. However, ALS are also formed by glycosylase-independent routes. AP-sites are formed by spontaneous base loss and are further processed to SSBs by AP-endonucleases. In addition SSBs are formed by oxidative attack at the sugar-phosphate backbone or as intermediates in other DNA-processing pathways, including repair of ICLs. An alternative or additional explanation to the reduced levels of ALS in the resistant cells could thus be increased processing of AP sites and SSBs. Notably, we did not detect any overt changes in expression of proteins involved in damage-general steps of BER/SSBR, including APE1, POLB, PCNA, LIG1, and LIG3 (Fig. 2). One exception was a modestly increased level of the scaffolding protein XRCC1. However, whereas the total level of the nick-sensing enzyme Poly(ADP-ribose) polymerase 1 (PARP-1) was similar in the two cell lines, a high molecular weight (HMW) form apparently constituting autoribosylated and activated PARP-1 [55] was observed exclusively in the resistant cells, even after treatment with 50 µM Melphalan (Fig. 5B, upper and middle panels). To further substantiate the activation of PARP-1 by autoribosylation, we treated the cells with 4-amino-1,8-naphthalimide (4-AN), a potent inhibitor of PARP-1 and of SSB repair [56]. This mediated a complete loss of the HMW (PARylated) form and a concomitant increase in the faster migrating unmodified PARP-1 (Fig. 5C), in agreement with loss of autoribosylation. The markedly increased PARP-1 activation was somewhat surprising given the lower level of PARP-1 trigger-lesions (ALS) in the resistant cells and suggests that the majority of these sites are efficiently repaired in the resistant cells. Notably, the increased enzymatic activity of PARP-1 in the resistant cells conforms to the upregulated level of c-MYC (Fig. 2). c-MYC was recently demonstrated to regulate the activity of PARP-1 via downregulation of the PARP-1 inhibitor protein BIN1, thereby increasing Cisplatin resistance [57].

To test whether increased PARP-1 activation also mediated altered sensitivity to PARP-1 inhibition, we next treated both cell lines with 4-AN and subjected the cells to MTT assay. As illustrated in Fig. 5D (upper panel), the Melphalan-resistant cells were significantly more sensitive to PARP-1 inhibition than the parental cell line. The increased sensitivity of Melphalan-resistant cells to PARP-1 inhibition was also observed when using another PARP-1 inhibitor, KU58684 (Fig. 5D, lower panel).

In summary, this suggests that the Melphalan-resistant cells have a lower level of ALS that may impede productive processing of Melphalan-ICLs. The lower level of ALS is apparently mediated both by reduced formation of AP-sites by glycosylases and by improved PARP-1- induced processing of ALS as substantiated by the unique sensitivity of the resistant cells to blockage of SSB repair. However, PARP-1 is also involved in other cellular defense mechanisms such as repair of DSBs and an effect of PARP-1 inhibition via such alternative routes could thus not be excluded.

### Proteins Involved in NHEJ are Up-regulated in Melphalan Resistant Cells

**Figure 6:**
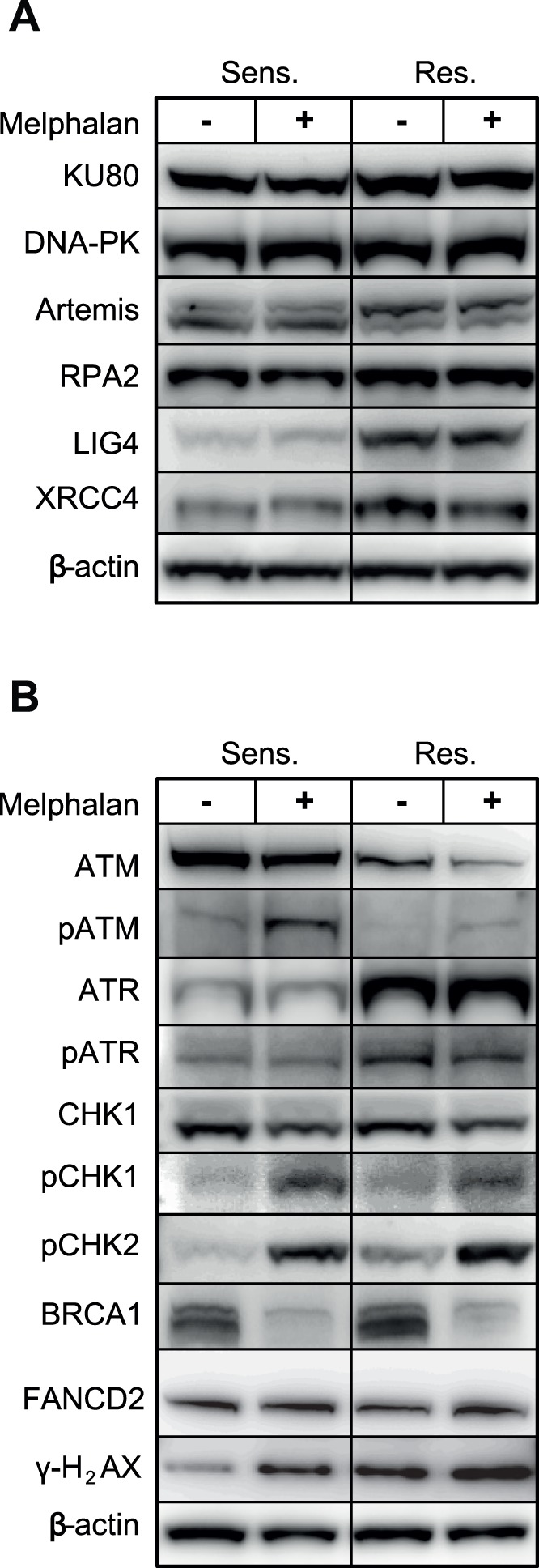
Level of DNA repair proteins (A) and damage signaling kinases (B) involved in DSB repair in Melphalan-sensitive and -resistant cells. (A) Quantitative western blot analysis revealed that the NHEJ proteins DNA ligase IV, XRCC4 and RPA2 are significantly and constitutively upregulated in Melphalan-resistant cells. In addition, KU80 and phosphorylated Artemis was consistently, although not significantly upregulated. Exposure to high dose Melphalan did not mediate further alteration of NHEJ proteins in the cell lines. (B) The steady-state levels of the kinase ATR and its phosphorylated form are increased in Melphalan resistant cells. Conversely, ATM is constitutively downregulated in the same cells. Rather than activation of CHK1, increased steady-state phosphorylation of pCHK2 was observed in resistant cells. Notably, high dose Melphalan strongly activates CHK2 in both sensitive and resistant cells but was consistently higher in resistant cells. Moreover, BRCA1, which is slightly induced in resistant cells, becomes markedly reduced in both sensitive and resistant cells after high dose Melphalan indicating temporary downregulation of HRR. Also note the the lack of apparent monoubiquitinylation of FANCD2 (B). β-actin was used as reference for quantitative analysis.

DNA DSBs are repaired via two major pathways; homologous recombination repair (HRR), and non-homologous end-joining (NHEJ) [58]. HRR takes advantage of homologous DNA templates present in late S and G2 and is thus error-free. NHEJ simply re-joins broken DNA ends, but is error-prone since it often involves processing of the ends to produce ligatable ends. NHEJ may thus result in chromosomal translocations, which are commonly observed in multiple myeloma. NHEJ may operate throughout the entire cell-cycle and appears to be the major route for DSBs repair in vertebrates. The classical NHEJ pathway is initiated by binding of the KU heterodimer to the ends of a DSB, and subsequent recruitment of the catalytic subunit of DNA-PK (DNA-PKcs) to form the active holoenzyme. DNA-PK_CS_ is autophosphorylated and also phosphorylates other NHEJ proteins such as RPA2, WRN, XLF, LIG4, XRCC4 and Artemis to facilitate end-joining and repair ([59] and references therein). More recently alternative NHEJ pathways have been described, termed Alt-NHEJ, which is dominated by microhomology-mediated end-joining [58]. Alt-NHEJ is mediated by PARP-1/2, and involves WRN and ligation by LIG3/XRCC1. Interestingly, the protein quantitation experiments indicate that several factors of the classical NHEJ pathway are upregulated in the Melphalan-resistant cells (Fig. 2 and 6A), with LIG4 (2.51±0.08, p = 0.001), XRCC4 (1.69±0.19, p = 0.02), and RPA2 (1.69±0.0.34, p = 0.03) showing significant upregulation. Artemis, which is involved in DNA end-processing prior to ligation, is present in two forms in both parental and resistant cells. Notably, however, a marked shift from the low-MW form conforming to unphosphorylated Artemis, to the high-MW form conforming to phosphorylated (activated) Artemis was observed in the resistant cells (Fig. 6A). These results indicate that the final steps of NHEJ encompassing end-processing and ligation are upregulated in the Melphalan-resistant cells.

No marked alteration in the steady-state level of most proteins involved in HRR (RAD50, RAD51, RAD52, MRE11) was observed in the Melphalan-resistant cells (Fig. 2). One exception was, however, a moderate increase in BRCA1 (1.43±0.10). However, subsequent to high dose Melphalan, the level of BRCA1 was reduced in both the sensitive (0.54±0.32) and resistant (0.56±0.11) cells, suggesting a down-regulation of HRR during high-dose treatment (Fig. 6B). In summary, these results indicate that upregulation of NHEJ, including a potential involvement of PARP-1 to facilitate synapsis of DSBs [60], may contribute to the Melphalan-resistant phenotype.

### ATM and ATR Signaling Pathways are Modulated in Melphalan Resistant Cells

The apparent switch in the levels of NHEJ versus HRR prompted us to investigate the involvement the damage-signaling kinases involved in DSB repair. DSB repair is initiated by three kinases belonging to the family of phosphatidylinositol 3-kinase-related kinases (PIKKs); DNA-PK, ATM (ataxia telangiectasia mutated) and ATR (ataxia telangiectasia mutated) [61]. While DNA-PK and ATM primarily respond to DSBs induced by e.g. ionizing radiation, ATR responds to replication blocks or other factors that induce extended stretches of ssDNA. Recent research has, however, demonstrated that the ATM- and ATR-initiated pathways are not separated, but highly interconnected and that ATM and ATR in fact may directly interact and activate each other [62].

Whereas no difference in the level of DNA-PKcs was observed in the resistant and sensitive cells (Fig. 6A), marked alterations were observed for ATM and ATR. As illustrated in Fig. 6B, the steady state-levels of ATM were significantly higher in the Melphalan-sensitive cells, whereas this was reversed for ATR. Moreover, subsequent to high-dose Melphalan, substantially more activated ATM (pS1981) was observed in the sensitive cells, whereas the level of activated ATR (pS428) was highest in the resistant cells. The increased CHK2-activation in the resistant cell line was also intriguing, since CHK2 is generally regarded as a tumor suppressor mediating cell cycle delay/arrest, apoptosis and DNA repair and is a canonical target of ATM [63]. Likely, an effect of CHK2 is not mediated via phosphorylation of p53, since p53 is mutated in RPMI8226 cells [64], as is also substantiated by the reduced level of p21 in the resistant cells (Fig. 2). Furthermore, this would have resulted in increased cell-cycle arrest and apoptosis in the resistant cells, which is not substantiated by the cell cycle analyses (Fig. 1B), the lack of modulation of pro- (BAX) and anti- (BCL2) apoptotic proteins (Fig. 2) and the low degree of PARP-1 cleavage (Fig. 3B).

**Figure 7:**
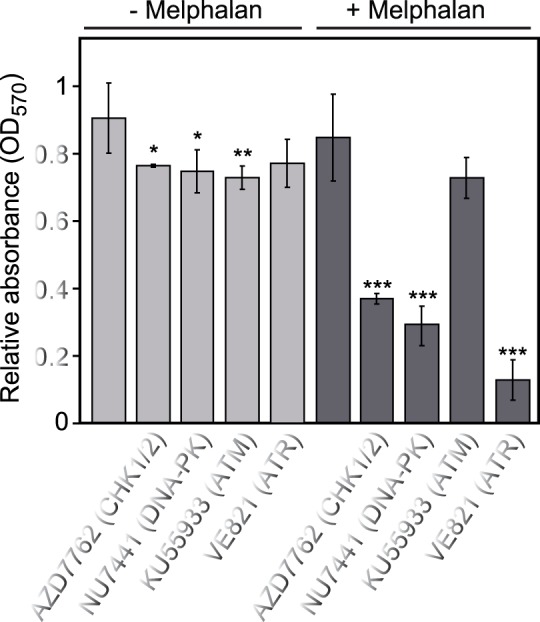
Effect of PI3-kinase-like kinase (PIKK)- and CHK1/2 inhibitors on the proliferation of 8226-LR5 cells. The MTT assay was used to monitor the survival after 72h incubation with the various inhibitors ±2.5 Melphalan. CHK1/2 inhibitor AZD7762 (0.1 µM), DNA-PK inhibitor NU7441 (0.2 µM), ATM inhibitor KU55933 (3 µM), ATR inhibitor VE821 (1 µM). Whereas a moderate inhibition on proliferation was observed with either inhibitor alone, strong inhibition was observed in combination with melphalan for the CHK1/2, DNA-PK and ATR inhibitors. Each bar represents the mean of at least 5 independent experiments with standard deviations as indicated. P-values were calculated using 2-sample t-test with equal variance. >95%, >99% and >99.9% confidence levels indicated by *, **, and ***, respectively.

To gain further insight in the relative roles of PI3-kinase-like kinases (PIKKs) as well as the downstream kinases CHK1/2 in mediating Melphalan resistance, we treated the resistant cells with varying concentrations of inhibitors against ATR (VE821), ATM (KU55933), DNA-PK (NU7441) and CHK1/2 (AZD7762) in the presence- or absence of Melphalan. Whereas a modestly decreased proliferation was observed with each of the inhibitors in the absence of Melphalan, a marked inhibition was observed when either CHK1/2, DNA-PK or ATR was inhibited in the presence of Melphalan. Notably, inhibition of ATR caused the strongest inhibition of proliferation, whereas inhibition of ATM mediated no significant reduction in proliferation. This corroborates the marked shift in expression and activation of ATM and ATR in the resistant cells and strongly supports that ATR plays a significant role in mediating Melphalan resistance in our experimental system. Moreover, it is tempting to speculate that ATR may contribute to NHEJ activation via activation of DNA-PK. A function of DNA-PK downstream of ATR would also conform to the lower effect of DNA-PK- compared to ATR inhibition on Melphalan sensitivity (Fig. 7). Notably ATR-dependent activation of DNA-PK has previously been observed subsequent to UV-induced replication stress in HeLa cells and this activation was mediated by phosphorylations on DNA-PK distinct from those observed subsequent to ionizing radiation [65]. Moreover, DNA-PK has been shown to phosphorylate CHK2 [66]. Although we did not observe Melphalan-induced phosphorylation of DNA-PK by using a phospho-specific pT2609 antibody (data not shown), potential activation of DNA-PK and NHEJ via ATR as well as the individual roles of CHK1/2 in mediating Melphalan resistance clearly warrants further investigation.

### Phosphorylated γH2AX foci are Formed more Rapidly and Persist for a Shorter Time in Melphalan Resistant 8226 Cells

**Figure 8:**
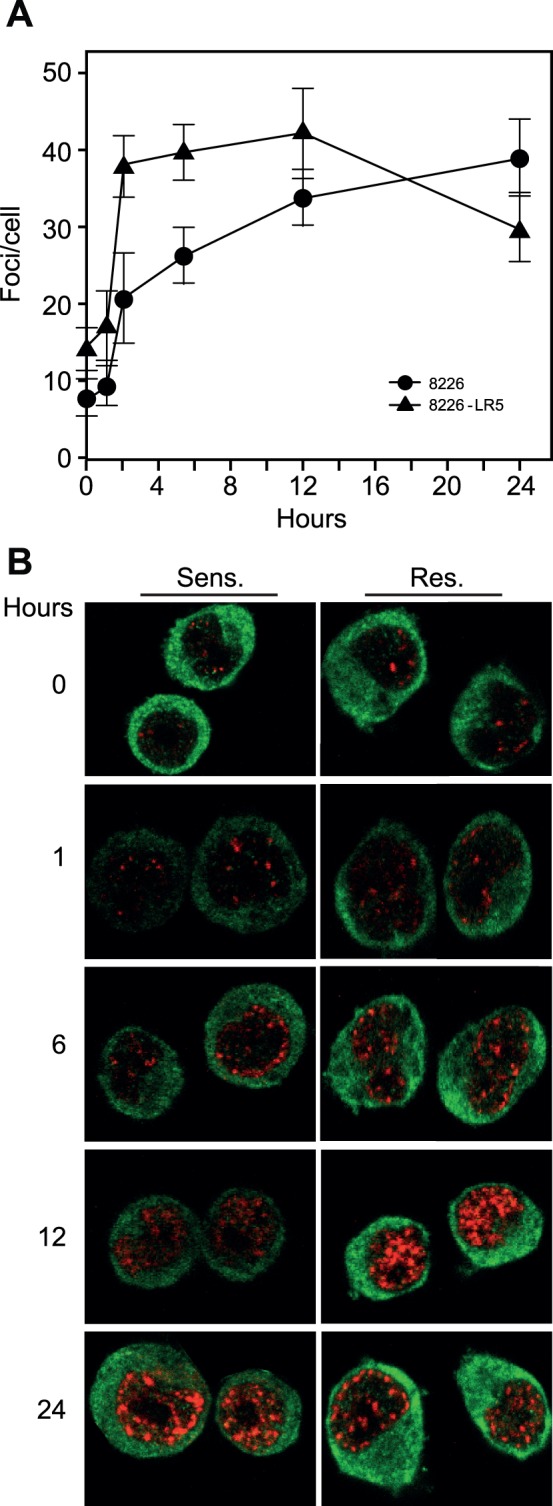
Melphalan-resistant cells have a more rapid kinetics of γH2AX foci formation than sensitive cells. (A) The number of γ-H2AX foci per cell nucleus (N>13) was manually quantified for each time point and the experiment was performed in triplicate. Corresponding standard deviation bars are indicated. P<0.01 at all time points as calculated using 2-sample t-test with equal variance. (B) Representative confocal images of the cells at different time points subsequent to Melphalan treatment. Foci formation was monitored using γH2AX polyclonal antibody (red) and due to the significant fraction of NFkB protein present in the cytoplasm of these cells, NFkB p65 mouse monoclonal antibody was used as control for cytoplasmic staining (green).

The level of nuclear foci of γH2AX is generally used to monitor the number of DSBs that are marked for processing subsequent to genotoxic stress and is also a sensitive marker of DNA damage induced by ICL-inducing agents [67]. To monitor a potential differential formation of- and persistence of such foci in the two cell lines, cells were fixed at different time points subsequent to treatment with 25 µM Melphalan, incubated with γH2AX antibodies, and nuclear γH2AX quantified subsequent to confocal analysis. Due to the half-life of Melphalan in cell culture medium at 37 degrees (∼ 1 h), cell washing after pulse drug exposure was not performed [68], [69]. Notably, a significantly higher number of foci were observed in the non-treated, resistant cells (0 hours). Subsequent to Melphalan treatment, a markedly increased accumulation of γH2AX foci was observed at early time points in the resistant- compared to the sensitive cell line (Fig. 8 A,B). In the Melphalan-resistant cells the number of foci decreased at 24 h, whereas an increase in the number of foci was observed in the sensitive cells. These results are entirely in agreement with the proposed increased repair of Melphalan-induced DNA damage in the resistant cells.

### Concluding Remarks

The present study reveals several DNA damage response factors as novel potential contributors to Melphalan resistance. Our results are in agreement with recent findings that productive repair of Cisplatin-ICLs is suppressed by base excision repair intermediates in the vicinity of the Cisplatin-ICL [34]. Here we demonstrate that three DNA glycosylases, UNG2, NEIL1 and MPG are significantly downregulated in the Melphalan-resistant cells. This is further corroborated by a markedly reduced UNG enzyme activity as well as doubling of the genomic 8-oxodG content in the resistant cells. Moreover, the lowered expression of the glycosylases was accompanied by an increased tolerance towards agents inducing their respective DNA base substrates. This is in agreement with previous findings demonstrating that overexpression of MPG increases sensitivity towards temozolomide [70] as well as MMS [71]. The genomic accumulation of 8-oxodG was apparently not caused by an increased level of ROS in the resistant cells and did not change subsequent to high-dose Melphalan. Thus a more likely explanation is that the observed accumulation of 8-oxodG is mediated by reduced excision by DNA glycosylases. Notably, OGG1 is regarded the prime glycosylase for removal of genomic 8-oxoG, with NEIL1 as a ubiquitously expressed [72]. Since the OGG1 expression was barely above the detection level in both cell lines, the markedly increased 8-oxodG level is most likely mediated by the threefold reduced level of NEIL1 in the resistant cells. It is currently not clear whether the marked downregulation of UNG2 mediates a corresponding increase in overall genomic uracil. Furthermore, it remains to be elucidated whether preferential deamination of cytosine occurs in regions flanking Melphalan-ICLs similar to what is observed at Cisplatin-ICLs. To our knowledge, the three-dimensional structure of a DNA Melphalan-ICL has not been resolved. Whether this ICL mediates the same degree of single-stranded DNA and extrahelical bases flanking the lesion as seen in the cisplatin-ICL [73], thus remain to be investigated. This could certainly also impact the rate of oxidative base damage at Melphalan ICLs. In conclusion, downregulation of initiator BER glycosylases may provide a selective advantage by lowering the levels of ALS repair intermediates that would obstruct ICL processing. Reduced excision of base lesions such as uracil and 8-oxoG, which both *per se* are regarded non-cytotoxic, would also make biological sense from another point of view, by increasing the overall mutation frequency in the resistant cells. This would likely be beneficial under the selective pressure imposed by Melphalan therapy.

A second factor contributing to reduced steady state levels of ALS would be increased repair of such sites. Although we did not observe a general upregulation of factors involved in AP-site or SSB processing, the observation of PARP1 autoribosylation exclusively in the resistant cells strongly support that such factors are more efficiently recruited to ALS in the resistant cells. This was also corroborated by the increased sensitivity to PARP-1 inhibition of the resistant cell line. Whether this increased sensitivity is mediated exclusively via reduced SSB-repair in the cells, however, remains elusive, since PARP-1 is also involved in other processes including DSB repair by mediating synapsis of strand breaks in NHEJ [60]. A recent study also demonstrated that PARP-1 bound with higher affinity to Cisplatin-induced ICLs than to undamaged DNA [74], supporting that PARP-1 may be involved in the recruitment of factors contributing to ICL- or ICL-induced DSB processing. If this is so, co-treatment with PARP-1 inhibitors should certainly be investigated further as a means to overcome sensitivity to ICL-inducing agents by inducing symbiotic lethality.

Our data suggest the potential involvement of an ATR/DNA-PK/CHK2 pathway in the stress response triggered by Melphalan and underscores that the classical ATM-CHK2/ATR-CHK1 pathways are likely much more versatile and dynamic than previously anticipated. This is also substantiated by studies of cisplatin-induced cytotoxicity in renal cells, in which ATR, but not ATM and DNA-PK is specifically activated during Cisplatin treatment, and that this results in ATR-dependent phosphorylation of both CHK1 and CHK2 [75]. In the same study CHK1 was degraded via the proteosomal pathway following phosphorylation, whereas CHK2 remained activated. The authors also report that Cisplatin-induced p53 activation and apoptosis are suppressed in ATR-deficient fibroblasts. The lack of apparent apoptosis-induction in the RPMI8226 cells may be explained by the mutant p53 protein, and the ATR-CHK2 activation rather mediates increased efficiency in the repair of DSBs and/or modulated checkpoint signaling. Moreover our data also support that DNA-PK may be involved in CHK2 activation, in accordance with previous observations [66]. DNA-PK may thus have a dual function in the cells in mediating CHK2 activation (together with ATR) and in initiating NHEJ. Upregulation of several proteins involved in the classical NHEJ pathway could then contribute to the observed increased efficiency in the repair DSBs, potentially involving increased DSB synapsis by autoribosylated PARP-1 [60].

In summary, we have identified several novel candidate DNA damage response proteins that may contribute to development of Melphalan resistance. We will now include these candidate proteins in SID-MRM-based targeted quantitative proteomics analyses in a larger patient cohort to determine their value in overcoming drug resistance in multiple myeloma, either as targets for substitution therapies or adjuvants for existing therapies.

## Materials and Methods

### Cell Lines

Multiple myeloma cell lines 8226 and its Melphalan resistant derivative 8226-LR5 [15], were kindly provided by Prof. William S. Dalton at the H. Lee Moffitt Cancer Center & Research Institute at the University of South Florida. Cells were cultured in RPMI 1640 (Sigma Aldrich) medium containing 10% (v/v) FBS (GIBCO) heated for 30 minutes at 56°C, 1% (v/v) of 200 mM L-glutamine (Lonza), 1% (v/v) of 10 mg/mL gentamicin (GIBCO) and 1% (v/v) of 250 UG/mL Amphotericin B (GIBCO). Melphalan resistant 8226-LR5 cells were maintained under constant selection through the addition of 1 µM Melphalan (Sigma-Aldrich, St. Louis, MO) twice per week. Subsequent to addition of the maintenance Melphalan, 8226-LR5 cells were allowed to grow at least for 3 days prior to any additional treatment or cell extract preparation.

### Cell Treatment with Melphalan and Preparation of Cell Extracts

Cells were treated with 50 µM Melphalan or vehicle control (acidified ethanol) for 6 hours in a ratio 1.10^6^/mL, harvested and washed 3 times with PBS. Cells were resuspended in buffer 1 containing 10 mM Tris-HCl pH 8.0, 200 mM KCl and diluted in the same volume (cells+buffer 1) of buffer 2 (10 mM Tris-HCl pH 8.0, 200 mM KCl, 2 mM EDTA, 40% glycerol, 0.5% NP40, 1 mM DTT, 1% phosphatase inhibitor cocktails 1 and 3 (Sigma), and 2% Complete EDTA-free (Roche)). After incubation for 1.5 h at 4°C, cell extracts were sonicated for 2 min, following centrifugation at 14,000 rpm for 10 min. Supernatants were collected and used for western blot analysis. Protein concentration was determined by using the BioRad protein assay (BioRad laboratories).

### Western Blot Analysis

Proteins were heated in LDS loading buffer (Invitrogen) prior to separation on pre-cast 4–12% denaturing NuPAGE gels using MOPS run buffer (Invitrogen) and electroblotting to PVDF membranes (Immobilon, Millipore) for 1∶30 h at 30 V. Western analysis was performed using the antibodies targeted against the following (dilutions or concentrations of antibodies are indicated): Artemis (ab35649, 1∶500), ATR (ab10312, 1∶15000), Chk1 (ab2845, 1∶3000 dilution), Cdc5L (ab51320, 1∶100), p21 (ab18209, 0.5 µg/mL), γ-H2AX (ab2893, 1∶1000), 14-3-3β (ab16859, 1∶2000), PRP19 (ab27692, 1 µg/mL), DNA polymerase β (ab3181, 1∶200), PCNA (ab29, 0.5 µg/mL), KU80 (ab3107, 0.5 µg/mL), MSH2 (ab16833-50, 1 µg/mL) DNA ligase 1 (ab615, 1∶3000), DNA ligase 3 (ab587, 2 µg/mL) and XPA (ab65963, 1 µg/mL), MPG (ab55461, 1 µg/mL) from Abcam; pATM (4526S, 1∶1000), pATR (2853, 1∶1000), pChk2 (2661, 1∶1000), pS317Chk1 (2344, 1∶1000), pS345Chk1 (2348, 1∶1000), Cdc25B (9525, 1∶1000), Cdc25C (4688, 1∶1000), pCdk1 (9111, 1∶1000) and BAX (2772, 1∶1000) from Cell Signaling; Cdk2 (sc-748, 1∶1000), c-Myc (sc-42, 1∶500), Bcl-2 (sc-65392, 1∶1000), MLH1 (sc-56159, 1∶1000), XRCC1 (sc-11429, 1∶500), ERCC1 (sc-10785, 1∶1000), FANCD2 (sc-20022, 1∶1000), DNA-PK (sc-9051, 1∶1000), MBD4 (sc-10753, 1∶1000), MRE11 (sc-5859, 1∶500), Rad50 (sc-20155, 1∶1000), Rad51 (sc-8349, 1∶1000), Rad52 (sc-8350, 1∶1000), RPA2 (sc-53496, 1∶1000) from Santa Cruz; ATM (A300-299A, 1∶5000), BRCA1 (A300-000A, 1∶5000), FEN1 (A300-256A, 1∶10000) from Bethyl laboratories; p53 (MS-105-p1, 0.5 µg/mL) from Neo markers; PARP-1 (04–575, 1∶1000) from Millipore; poly(ADP-ribose) from Trevigen (4335, 1∶1000) DNA ligase 4 (HPA001334, 1∶1000) from Sigma; XRCC4 (GTX109632, 1∶1000) from Gene Tex; CDC4 (39–5800, 0.5 µg/mL) from Zymed; 14-3-3σ (H00002810, 1∶1000), TDG (H00006996, 1∶1000) and CDK1 (HPA003387, 1∶1000) from Abnova; WEE1 (AP8106b, 0.5 µg/mL) from Abgent. The polyclonal antibodies PAPE1 and PU059 recognizing APE1 and the catalytic domain of UNG, respectively, were generated in our own lab (0.5 µg/mL dilution). The NEIL-1 and OGG1 polyclonal antibodies were kindly provided by Prof. Magnar Bjørås at the University of Oslo. Anti-β-actin mouse monoclonal antibody (ab8226, Abcam, 1∶10000) was used to normalize the data. After incubation with HRP-conjugated secondary antibodies (Dako Denmark, 1∶5000), membranes were incubated with SuperSignal West Femto Maximum Sensitivity Substrate (Thermo Scientific) and bands were detected on a digital imaging system IS4000R Kodak (Fisher Scientific). One exception was parallel detection of PARP-1/PARylated PARP-1, which was performed on the Odyssey platform (Li-Cor Biosciences – GmbH) subsequent to incubation with Goat anti-mouse IRDye800LT and goat anti-rabbit IRDye680LT secondary antibodies (Li-Cor Biosciences). Quantitative analysis was performed using 3 to 5 biological replicates, each consisting of a set of sensitive and resistant cells both at normal growth and subsequent to treatment with high dose Melphalan for 6 hours. Band densities of target proteins were measured for each replicate by using the Kodak Molecular Imaging software version 4.0.1. After subtracting background intensity values, densities of target proteins of each replicate were normalized according to -β-actin or -β-tubulin densities. After normalization, ratios of band densities of target proteins in resistant untreated cell extract versus sensitive untreated cell extract were calculated. The mean of 3 to 5 measurements of ratios Rc/Sc was calculated and the correspondent standard deviations were obtained using the following formula: 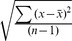, where x is the sample mean average and n is the sample size, described as STEDVA function in Excel.

### Flow Cytometry

For FACS analysis, one million cells were fixed in ice-cold 100% methanol and stored at 4°C until DNA measurement. The cells were washed with cold phosphate-buffered saline (PBS) and incubated with 200 µl of DNase-free RNAse A in PBS (100 µg/ml) for 30 min at 37°C before DNA staining with 200 µl of propidium iodide (50 µg/ml) at 37°C for 30 min. Cell cycle analyses were performed by using a BD FACSAria cell sorter (BD Biosciences). Propidium iodide stained cells were analysed at 488nm excitation (blue laser) and 575 nm emission. Cell cycle fractions were determined by using the BD FACSDiva software (BD Biosciences).

### Viability Assays

The 3-(4, 5-Dimethyl-2-thiazolyl)-2, 5-diphenyl-2H-tetrazolium bromide (MTT) assay was performed using Melphalan, methyl methanesulfonate (MMS), H_2_O_2_, 5-Fluorouracil, Mitomycin C (MMC) and 4-Amino-1,8-naphthalimide (4-AN), ATM inhibitor (KU55933) from Santa Cruz ATR inhibitor (VE821) from Axon Medchem, and DNA-PK inhibitor (NU7441) and CHK1/2 inhibitor (AZD7762) from Selleckchem. The PARP1-inhibitor KU58684 (Kudos/AstraZeneca) was kindly provided by Dr. Fran*ç*oise Dantzer at the University of Strasbourg. Exposure to UVB (312 nm) was performed using ultraviolet lamp from Vilber Lourmat. Briefly, myeloma cells resuspended in RPMI 1640 medium were added to 96-well plates (1×10^4^ cells/100 µl/well), and then exposed to varying concentrations of agents (100 µl/well). Drugs used in the cytotoxicity assays were used at concentrations in the same range as previously reported IC_50_ values in RPMI8226 for 5-FU [76] and H_2_O_2_ [77], and in lymphocytes for MMS and MMC [78]. Cells exposed to ultraviolet radiation were resuspended in PBS instead of RPMI medium prior to addition in 96-well plates (2×10^4^ cells/50 µl/well). After irradiation with 100 J/m^2^ UVB, 150 µl of RPMI medium were added to each well to allow cell proliferation. Wells containing only medium and medium with a particular agent were also included as controls. Results were measured at 0, 48, 72 and 96 hours after treatment. After each time point, 100 µl were removed from each well and 100 µl of 1 mg/ml MTT were added to each well, followed by incubation at 37°C in culture hood for 4 hours. Subsequently, 130 µl were carefully removed from each well and acidified isopropanol (100 µl) was added to the plates. Plates were covered with tin foil and agitated in orbital shaker for 1 hour prior to absorbance measurement in a Fluostar optima plate reader (BMG labtech). Measurements were performed at 590 nm with a reference filter of 620 nm. Standard deviations for each time point are indicated.

### UDG Assay

Uracil DNA glycosylase activity was measured against [^3^H]dUMP-containing calf thymus DNA (U:A) as described (Kavli et al, 2002). Briefly, cell extracts were diluted in assay buffer (20 mM Tris-HCl pH 7.5, 60 mM NaCl, 1 mM DTT, 1 mM EDTA and 7.5 mM MgCl_2_) and incubated at 30°C for 10 minutes with ss- or dsDNA substrate. The reaction was quenched by addition of 50 µl salmon DNA and 500 µl of 5% TCA following centrifugation at 14,000 rpm for 10 minutes at 4°C. The supernatant was analyzed by liquid scintillation counting. Experiments to monitor residual UDG activity in cell extracts were performed by incubating cell extracts with the UNG inhibitor Ugi on ice for 15 minutes prior to addition of substrate.

### Genomic 8-oxodG Analysis

Genomic DNA was isolated using the DNeasy® Blood and Tissue kit (Qiagen, Hilden, Germany) according to the manufacturer’s instructions. The kit's elution buffer contains EDTA, which inhibits nucleases during sample preparation for 8-oxo-dG measurement, so the final elution step was performed using 150 µl water instead of the provided elution buffer. The DNA was then enzymatically hydrolyzed to deoxynucleosides. To this end, 0.5 µg DNA was added to 40 µl of 100 mM NH_4_HCO_3_, pH 7.6, 1 mM CaCl_3_, 10 mM MgCl_2_, 1 U recombinant DNase I (Roche), 0.2 mU phosphodiesterase I from *Crotalus adamanteus* venom (Sigma-Aldrich), 0.1 U alkaline phosphatase from bovine intestinal mucosa (Sigma-Aldrich), 1.25 pmol [^15^N_5_]8-oxodG (Cambridge Isotope Laboratories) and incubated at 37 °C for 6 h. To precipitate contaminants that could potentially clog the HPLC column, five volumes of ice-cold methanol were added to the samples, mixed by vortexing, and centrifuged at 16,100 x g for 20 min at 4°C. The supernatants were transferred to new tubes and vacuum centrifuged at room temperature until dry. The resulting pellets were dissolved in 25 µl 5% methanol. Liquid chromatography/tandem mass spectrometric analysis was performed using an LC-20AD HPLC system (Shimadzu Corporation, Kyoto, Japan) coupled to an API 5000 triple-quadrupole mass spectrometer (Applied Biosystems, Carlsbad, CA, USA). The HPLC column was a Zorbax SB-C18 reverse phase chromatography column (2.1×150 mm, i.d., 3.5µm, Agilent Technologies, Santa Clara, CA, USA), protected with a Zorbax Reliance guard-column (4.6 mm×12.5 mm, Agilent Technologies) and the injection volume was 20 µl. The gradient used consisted of solvent A (water, 0.1% formic acid) and B (methanol, 0.1% formic acid) starting at 5% B for 0.5 min, ramping to 90% B over 6 min, holding at 90% B for 1.5 min and re-equilibrating with 5% B for 5 min at a flow rate of 300 µl/min. Mass spectrometric detection was performed using positive electrospray ionization in multiple reaction monitoring mode, monitoring the mass transitions 284.1/168.2 and 289.2/173.1 for 8-oxodG and [^15^N_5_]8-oxodG, respectively.

### Comet Assay

Cells (1 million cells/mL RPMI medium) were treated with different doses of Melphalan (50 µM and 100 µM) or vehicle control (acidified ethanol) for 6 h. The cells were harvested by centrifugation, embedded in 1% low-melt agar (LMA) and mounted on microscope slides. The embedded cells were lysed overnight at 4°C in lysis solution, treated 30 min in alkaline running buffer (pH>13.3) and subjected to electrophoresis as described [79]. DNA was visualized by ethidium bromide, and 100 comets were randomly selected and evaluated using Komet 5.0 imaging software (Andor Technology).

### Immunofluorescence

Cells were treated with 25 µM Melphalan for different time points and then fixed in freshly prepared 4% paraformaldehyde for 10 minutes on ice prior to addition of cold methanol (−20°C). After incubation for 20 minutes at −20°C, cells were washed three times with 2% FCS in PBS and incubated with anti-γ-H2AX rabbit polyclonal (ab11174, Abcam, 1∶200 dilution) and anti-NFkB p65 mouse monoclonal antibodies (sc-8008×, Santa Cruz, 1∶200 dilution) following overnight incubation at 4°C. Anti-NFkB p65 mouse monoclonal antibody was chosen as control for cytoplasmic staining, since the portion of NFkB present in the cytoplasm is easily visualized. Cells were then incubated with Alexa Fluor 647 goat anti–rabbit and 532 goat anti-mouse secondary antibodies (Invitrogen, 1∶4000 dilution) for 1 h at 37°C. Immunofluorescence data were acquired in a laser-scanning microscope (LSM 510 Meta; Carl Zeiss) equipped with a Plan Apochromat 63×1.4 NA oil immersion objective and images were analyzed using LSM 510 software (Carl Zeiss). No image processing except from contrast and intensity adjustments were performed.

## Supporting Information

Figure S1 **Intracellular level of ROS was detected by flow cytometry using 5-(and-6)-carboxy-2′,7′-dichlorodihydrofluorescein diacetate (carboxy-H2DCFDA, Molecular Probes) according to the manufacturer’s protocol.** Briefly, cells (1×10^6^ cells/mL) were washed with PBS and incubated in PBS buffer containing 10 uM dye for 30 min at 37°C. H2DCFDA-stained cells were returned to growth medium and treated with 50 uM Melphalan. Flow cytometry analysis was performed after 6 hours of incubation, at 488 nm excitation and 530 nm emission. Parallel treatment of cells with the oxidizing agent tert-butyl hydroperoxide (TBHP, Sigma) was included as a positive control for oxidative stress in the assay.
(EPS)
